# Editorial: Pre-and postharvest treatments with elicitors on the development of bioactive compounds and nutritional quality of fruit and vegetables

**DOI:** 10.3389/fnut.2022.1070945

**Published:** 2022-10-31

**Authors:** Fabián Guillén, Fariborz Habibi, John B. Golding

**Affiliations:** ^1^Department of Agro-Food Technology, University Miguel Hernández, Orihuela, Spain; ^2^Horticultural Sciences Department, University of Florida, Gainesville, FL, United States; ^3^New South Wales Department of Primary Industries, Ourimbah, NSW, Australia

**Keywords:** storage, quality, elicitors, bioactive compounds, fruit, vegetables, eco-friendly

## Introduction

Management factors in the field and the climate in which crops are grown have a strong effect on crop quality, including the nutrition and accumulation of bioactive compounds. In addition, different postharvest technologies are used to maintain product quality (including nutrition) through the supply chain. Although cold storage is a simple and widely used to maintain fruit quality, prolonged cold storage can result in the decline of organoleptic quality, nutritional values and levels of bioactive compounds in the fruit. In addition, cold storage can result in chilling injury and other physiological disorders (1).

This Research Topic is of importance to both human nutritionists and industry as it provides novel information on the implementation of elicitation strategies to increase the content of bioactive compounds and nutritional quality of fresh fruit and vegetables. Degreening with ethylene and application with other phytohormones and secondary metabolites implicated in plant systemic disease resistance have been revised in this Research Topic. In addition, new approaches which impact the accumulation of bioactive compounds as a result of different commercial and emerging postharvest technologies are described. Commercial postharvest technologies such as CO_2_ or 1-methylcyclopropene (1-MCP) treatment and emerging technologies based on inert gas applications such as cold atmospheric plasma (CAP) have been shown to result in improved nutritional quality.

## Postharvest treatments with phytohormones and secondary metabolites

The application of novel phytohormones as efficient elicitors to enhance quality and storability has been an active area of study. Phytohormones and secondary metabolites are the structures that play a major role in the plants surviving in their environment and overcoming stress conditions. All the phytohormones such as ethylene, gibberellins, melatonin (MT), salicylic acid (SA), and secondary metabolites such as glycine betaine (GB) have been studied under this Research Topic and have shown that the application of these treatments generally induce disease resistance and are involved in the elicitation of bioactive compounds and in the delaying of senescence. Chen et al. observed increased levels of anthocyanins in mangoes after ethylene treatments, suggesting that exogenous applications of ethylene might promote color changes by enhancing the activities of these pigment-metabolizing enzymes and increasing fruit ripening. This observation was supported by an upregulation of the relative expression of genes involved in ethylene biosynthesis contributing also to a higher expression of genes related to anthocyanin accumulation. However, increasing anthocyanins or antioxidant activity in fruit is not linked always to an increase in ripening or senescence. Habibi et al. studied the effect of GB increasing anthocyanin concentration or total polyphenol content in blood oranges. They also showed this treatment also delayed senescence during storage. This effect was observed through suppressed polyphenol oxidase activity but also stimulating the antioxidant enzyme activity system. In this sense, and following analysis undertaken by Zhang et al. in winter jujube, the antioxidant enzymatic system could be enhanced by the application of SA alone or in combination with 1-MCP and this treatment also delayed senescence. This is the first report in jujube showing the synergistic effect between two postharvest technologies in terms of enhancing fruit quality and elicitation of bioactive compounds including ascorbic acid or flavonoids. The authors also observed an increased content of soluble solids and total acidity in jujube fruit, an effect that was also observed also in blood oranges treated with GB. An increase in the levels of bioactive compounds, soluble solids, and organic acids impacts the nutritional and functional fruit quality. Accordingly, Bhardwaj et al. studied MT applications in mango fruit and showed this treatment alleviated chilling injury with a higher unsaturated/saturated fatty acid ratio. It was suggested that this helps preserve membrane integrity protecting the intracellular energy supply when fruits are stored at suboptimal temperatures. MT was shown to be a regulator of two intrinsically linked processes, ripening, and senescence. It is reported that ripening is positively regulated, while senescence is negatively regulated when these phytohormones are applied (2). Gibberellins are another well-known phytohormones which help protect membrane integrity (3). In this Research Topic, the mechanism affecting tuber dormancy regulation in potatoes was reported and showed that the application of gibberellins alone or in combination with other growth regulators has a significant effect on potato tuber dormancy. Postharvest dormancy is critical to the quality of this product during storage. The different potato genotypes studied displayed a significant positive correlation between dormancy and ABA levels and a negative correlation with respect to gibberellin content. This new information could be useful to develop storage strategies to maintain the nutritional and organoleptic quality of this product for a longer time without the use of chemicals.

## Other emerging postharvest technologies

This Research Topic compiled a range of different studies addressing emerging technologies affecting the accumulation of secondary metabolites. Secondary metabolites increase the plant defense system, aroma, and pigmentation of fruit and vegetables also affecting nutritional quality (4). In this sense, an emerging technology such as cold atmospheric plasma has shown to be highly effective. Jin et al. described the effect of this technology delaying fruit ripening and increasing total polyphenols and antioxidant activity in jujube fruit. These authors described genetic approaches to increased expression of different genes related to phenolic acid synthesis. Tilahun et al. applied already commercially available technologies such as CO_2_ and 1-MCP to peach fruit and these treatments not only increased total phenolics and flavonoids but also increased the antioxidant activity of the fruit. It has been suggested that the antioxidant balance is one of the main factors affecting a range of factors during storage and postharvest technologies which promote antioxidant activity can assist in delaying chilling injury and maintaining quality.

## Conclusions

In summary, the accumulation of secondary metabolites is regulated by complex signaling pathways involving phytohormones. The different technologies reported in this Research Topic have been shown to induce disease resistance in fruit and vegetables and are involved in the elicitation of bioactive compounds. These technologies have also been linked to a delay in fruit ripening and senescence, maintaining nutritional and functional quality during storage.

## Author contributions

All authors listed have made a substantial, direct, and intellectual contribution to the work and approved it for publication.

## Funding

Current work of the postharvest research group at University Miguel Hernández was funded by Conselleria d'Innovació Universitats, Ciència i Societat Digital (Generalitat Valenciana) through Prometeo Program (PROMETEO/2021/089). The Australian Citrus Postharvest Program (CT19003) funded by Horticulture Innovation and NSW Department of Primary Industries also contributed to this Research Topic.

## Conflict of interest

The authors declare that the research was conducted in the absence of any commercial or financial relationships that could be construed as a potential conflict of interest.

## Publisher's note

All claims expressed in this article are solely those of the authors and do not necessarily represent those of their affiliated organizations, or those of the publisher, the editors and the reviewers. Any product that may be evaluated in this article, or claim that may be made by its manufacturer, is not guaranteed or endorsed by the publisher.
